# Asymmetrical bilateral persistent sciatic artery complicated with right limb thrombosis: A rare CTA finding

**DOI:** 10.1016/j.radcr.2026.01.009

**Published:** 2026-02-04

**Authors:** Amir Hossein Mahdian Dehkordi, Mohsen Ghazi Soltani

**Affiliations:** a School of medicine, Kurdistan University of Medical Sciences, Sanandaj, Kurdistan, 6618634683, Iran; b Department of radiology, School of medicine, Kurdistan University of Medical Sciences, Sanandaj, Kurdistan, 6618634683, Iran

**Keywords:** Persistent sciatic artery, Thrombosis, Vascular anomaly, Computed tomography angiography, Bilateral, Limb ischemia

## Abstract

Persistent Sciatic Artery (PSA) is a rare congenital vascular anomaly resulting from the failure of the embryonic axial artery regression. Its bilateral occurrence is even rarer, carrying a risk of serious complications such as aneurysm formation or thrombosis. This report describes the case of bilateral PSA in a 67-year-old male with a history of diabetes and hypertension who presented with symptoms of intermittent claudication in his right lower limb. Computed Tomography Angiography (CTA) confirmed the presence of bilateral PSA and revealed a striking asymmetrical presentation. The right limb showed a ``complete'' PSA with a hypoplastic superficial femoral artery (SFA) and an extensive 33-cm intraluminal thrombosis. In contrast, the left limb demonstrated an ``incomplete'' PSA with a patent, dominant SFA. This case underscores the necessity of considering uncommon vascular abnormalities, especially their asymmetrical manifestations, in the differential diagnosis of lower limb ischemia. Furthermore, it highlights the critical role of CTA as the gold standard for accurate preoperative vascular mapping, since failing to identify this anatomical asymmetry may result in iatrogenic injury and inappropriate surgical planning.

## Introduction

The axial or sciatic artery is the main vascular supply to the embryonic lower limb. By around the 12th week of pregnancy or after the 22–25 mm embryonic stage, this primitive sciatic artery typically regresses as the femoral arterial system grows and becomes the main source of blood flow to the lower extremities [1,2].

A persistent sciatic artery (PSA) is a rare congenital vascular anomaly resulting from the failure of this normal embryological regression [3]. The estimated incidence of PSA is remarkably low, typically between 0.025% and 0.06% [4,5]. Its bilateral manifestation is even more uncommon, constituting only 12% to 32% of all reported cases [6,7].

Anatomically, PSA normally extends from the internal iliac artery, traveling along the sciatic nerve, through the greater sciatic foramen, and often joins or forms the popliteal artery. PSA is clinically significant due to its propensity for complications [4,5].

It is often asymptomatic until a complication develops. The most frequently reported complications are aneurysms (48% of cases), occlusion (9%) or thromboses (7%) [2,4]. The anomaly is commonly linked to aneurysm formation, with reported rates as high as 41–61 percent [6,8]. Other known side effects of PSA include atherosclerotic degeneration causing stenosis and critical limb ischemia due to thrombosis or distal thromboembolism [5].

This report details the case of a 67-year-old male with bilateral PSA who presented with critical limb ischemia due to unilateral long-segment thrombosis. We report this case to highlight the specific imaging features of this anomaly, particularly its asymmetrical presentation, and to emphasize the vital importance of a precise preoperative diagnosis.

## Case presentation

A 67-year-old male with a 12-year medical history of Type II Diabetes Mellitus and Hypertension presented with a 2-year history of progressive intermittent claudication (Fontaine Stage IIb). The patient reported a significant decrease in pain-free walking distance, primarily affecting the right calf, which had recently escalated to occasional rest pain (Fontaine Stage III).

Physical examination revealed chronic trophic changes in the right lower extremity, including mild gastrocnemius muscle atrophy and patchy alopecia of the pretibial region. There were no signs of active ulceration, gangrene, or dependent rubor. Vascular palpation demonstrated strong symmetrical femoral pulses bilaterally. However, a significant discrepancy was noted distally. While the left popliteal, Dorsalis pedis, and posterior tibial pulses were normal (2+), all pulses distal to the right femoral artery were clinically absent (0). The “Cowie sign” (a characteristic finding in PSA where the popliteal pulse is palpable despite an absent femoral pulse) was notably absent in this patient.

On bedside evaluation, the right foot appeared pale with a delayed capillary refill time of approximately 4 seconds, compared to 2 seconds on the left. Neurological examination showed intact sensation and motor function, ruling out acute sensory-motor deficit. Although formal Ankle-Brachial Index (ABI) manometry was not performed, the constellation of clinical findings (absent distal pulses, trophic skin changes, and prolonged capillary refill) strongly indicated advanced chronic limb-threatening ischemia (CLTI) on the right side.

Color and spectral Doppler sonography of the right dorsalis pedis and posterior tibialis arteries showed abnormal, low resistance monophasic waves with a peak systolic velocity (PSV) of 12 to 14 cm/s.

A 64-slice CT scanner was used for computed tomography angiography (CTA), which produced 3D visualizations and reconstructed images in the axial, sagittal, and coronal planes for additional assessment.

**Fig. 1:**
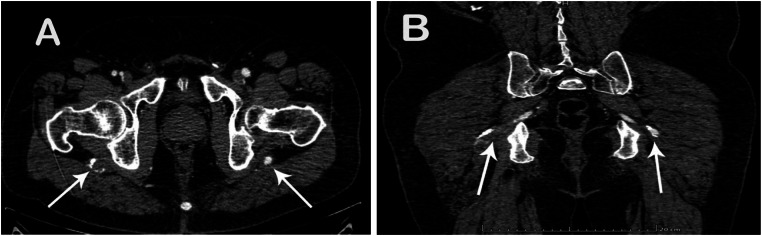
The Pelvic CTA of axial (A) and coronal (B) planes shows bilateral persistent sciatic artery (arrows) with calcified plaque and significant intraluminal thrombosis in right side.

**Fig. 2:**
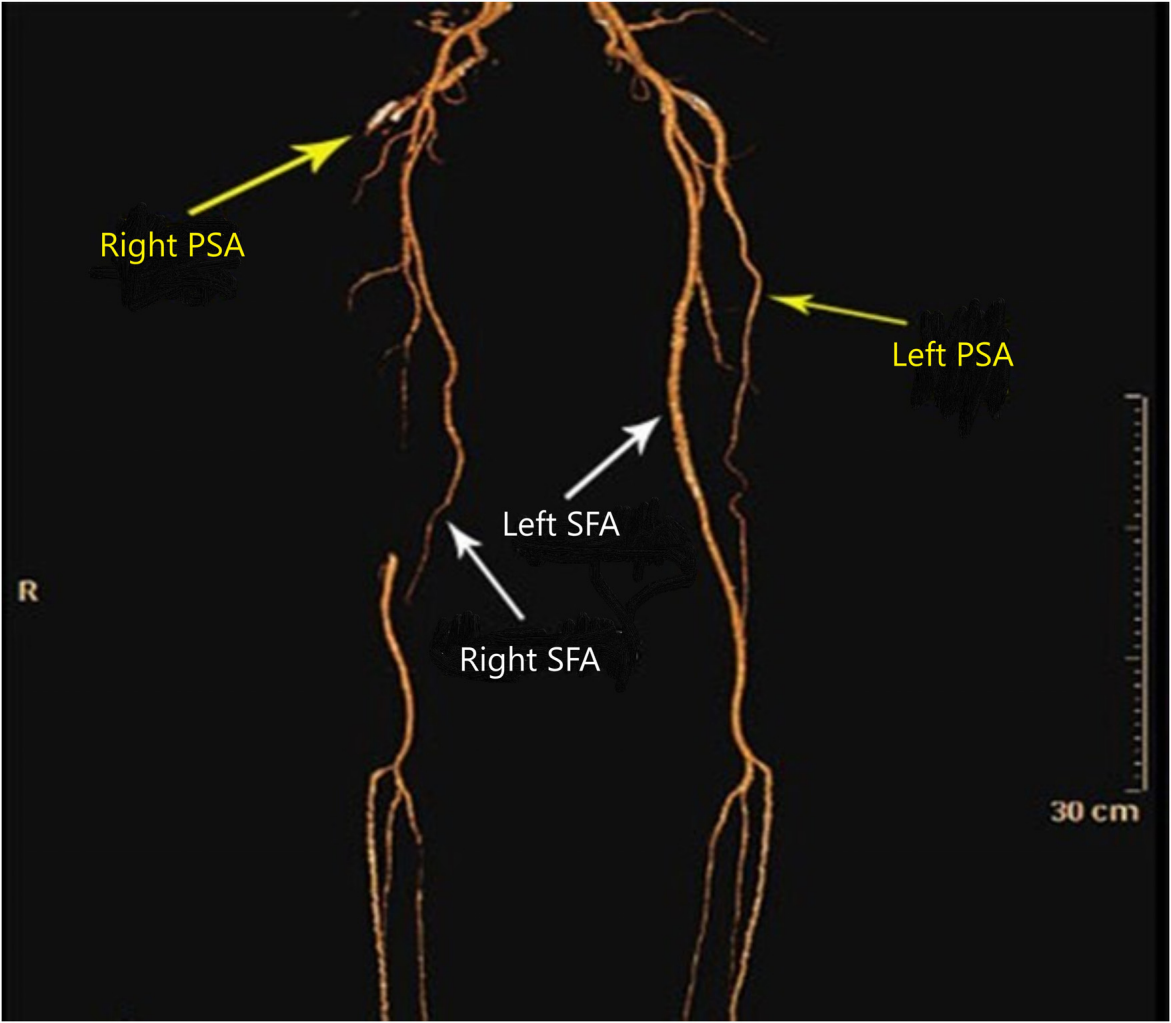
Anterior view of 3D CT reconstruction arteries. The CTA demonstrated right superficial femoral artery (SFA) was hypoplastic and no continuity to popliteal artery. The right PSA with calcified plaque and complete intraluminal thrombosis is noted. The left SFA have continuity with popliteal artery and the left persistent sciatic artery (PSA) extending downward and connected to left popliteal artery. Also, the bilateral persistent sciatic arteries are noted. (Yellow arrows).

**Fig. 3:**
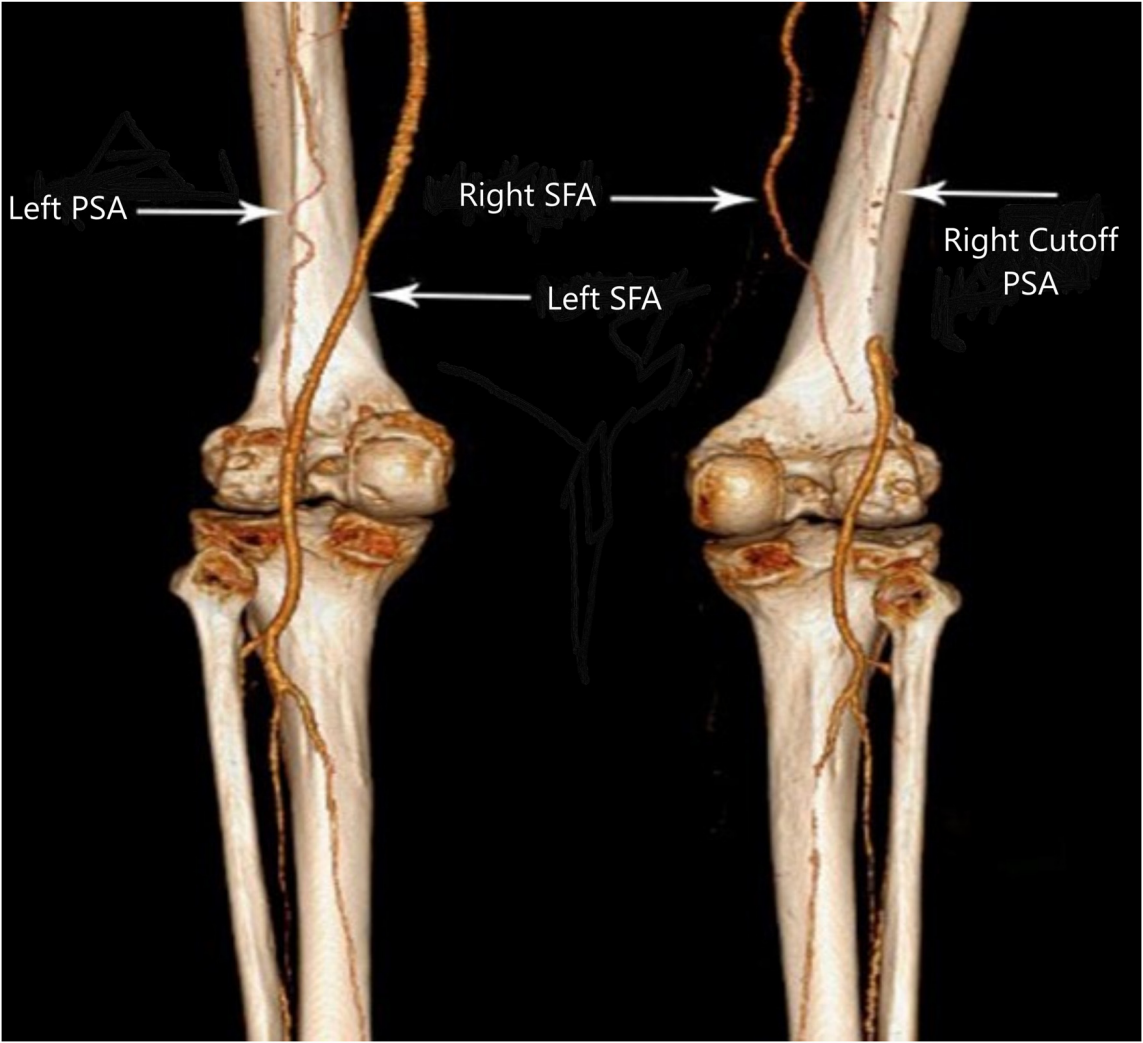
Posterior lateral view 3D CT reconstruction demonstrated right SFA have no continuity to popliteal artery but the left SFA have continuity to popliteal artery. The left PSA extending downward and connected to left popliteal artery. Also, the right PSA cutoff is seen.

The CTA demonstrated bilateral PSA (Fig. 1, Fig. 2). The right superficial femoral artery (SFA) was hypoplastic, terminating distally with no continuity to the popliteal artery (Fig. 2, Fig. 3).

**Fig. 4:**
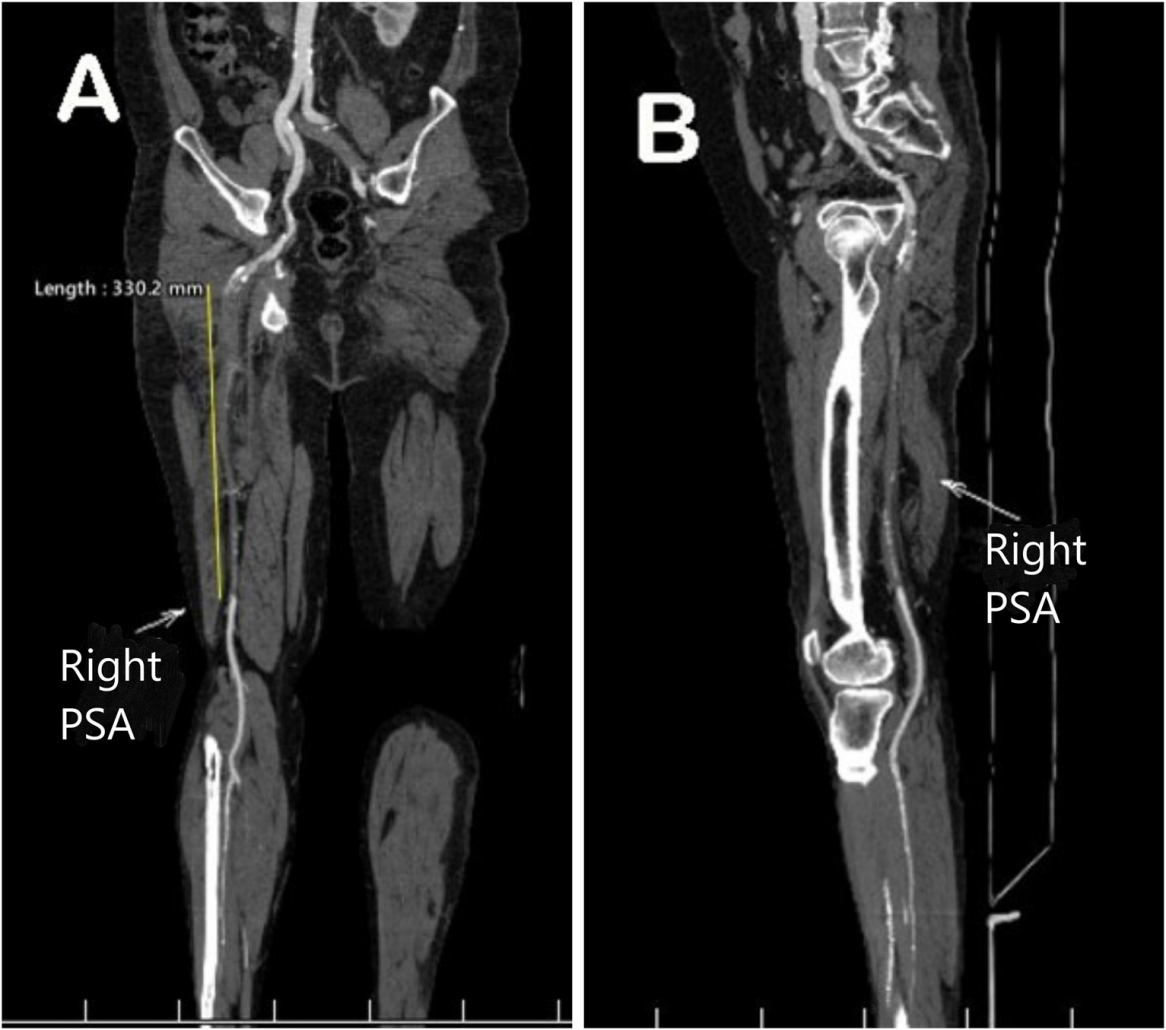
Coronal (A) and sagittal (B) CTA images of the right lower extremity. These images display the right PSA (arrows) with extensive intraluminal thrombosis measuring approximately 33 cm in length.

The right PSA extended to the popliteal artery but showed calcified plaque in the proximal part, leading to a complete intraluminal thrombus and cutoff artery. The occlusion measured approximately 33 cm in length (Fig. 4). Runoff was provided by several small collateral vessels.

On the left side, the SFA was normal, maintaining continuity with the popliteal artery, with no significant stenosis (Fig. 2, Fig. 3).

Following diagnosis, a vascular surgery consultation was requested.

## Discussion

### Diagnosis and classification

PSA is a rare embryological anomaly, with bilateral occurrence being even less common [2,9].

Physical examination findings such as the “Cowie sign” (palpable popliteal pulse with an absent femoral pulse), may be suggestive [2,10], but were absent in our patient.

Advanced imaging is necessary for the diagnosis of PSA, particularly bilateral forms [2,5]. While conventional angiography was historically the gold standard, CTA and Magnetic Resonance Angiography (MRA) are now preferred. These modalities offer superior anatomical detail of the PSA, its relationship to surrounding structures, the condition of the femoral system, and related complications (aneurysms or thrombosis) [4,10]. The diagnosis in this case was made using CTA and 3D CT reconstruction images.

**Fig. 5:**
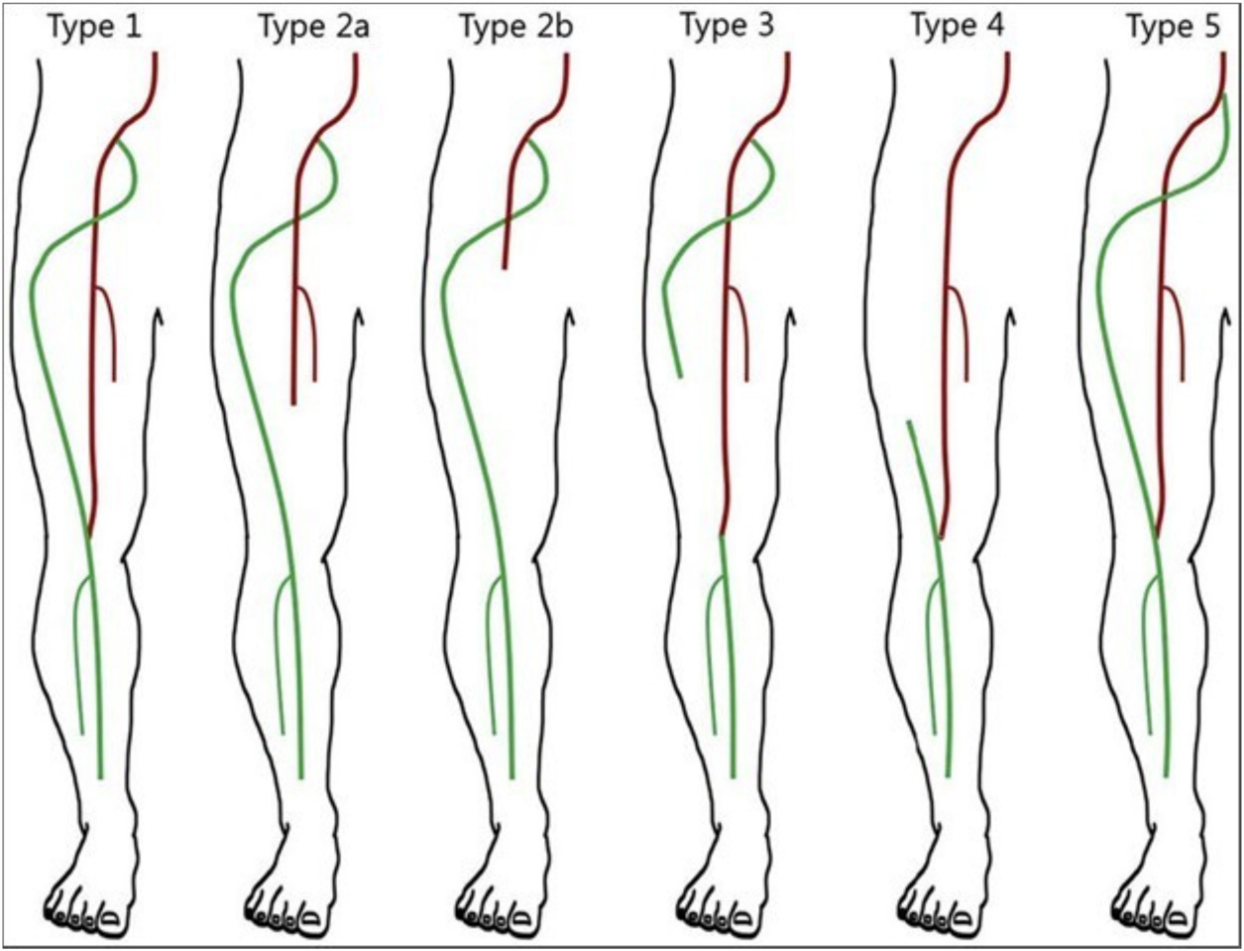
The Pillet et al. [12] classification (1980) of persistent sciatic artery. Red vessels represent the femoral system, while green vessels represent the sciatic system. This classification divides the anomaly into 5 main types based on the connection between the persistent sciatic artery and the superficial femoral artery (Adapted from Gauffre et al. [13]).

Several classification systems exist. According to the system by Bower et al. [11], a PSA is ``complete'' if it is the predominant arterial supply to the popliteal artery and the SFA is hypoplastic. It is ``incomplete'' if the SFA is the main blood supply to the popliteal artery and the PSA is less developed. Other classifications by Pillet et al. [12] and Gauffre et al. [13] also exist (Fig. 5).

Based on these systems, our patient's anatomy was classified as follows:

Right Limb: Complete type (Bower) or Type 2a (Pillet classification); the PSA was the dominant supply with a hypoplastic SFA.

Left Limb: Incomplete type (Bower) or Type 1 (Pillet classification); the SFA remained the dominant supply.

The primary diagnostic challenge of this report is the bilateral but asymmetrical nature of our patient's anatomy, which is classified as an incomplete PSA on the left (Type 1, Pillet) and a complete PSA on the right (Type 2a, Pillet). The presence of differing femoral and popliteal pulses in each limb, or the absence of a Cowie sign bilaterally, could easily misdirect the initial clinical assessment. This emphasizes the need for a high index of suspicion for asymmetrical bilateral PSA when evaluating lower limb ischemia.

### Clinical presentation and complications

PSA has a broad range of clinical manifestations from incidental findings [2] to severe ischemia that could endanger limbs [8,9].

The most common serious complication of PSA is aneurysmal degeneration, reported in up to 48-61% of PSA cases [6,8]. Aneurysms commonly occur in the gluteal segment and can lead to distal embolization, thrombosis, or compression of the sciatic nerve [5,6]. In our case, PSA aneurysm was not observed.

Other critical complications of PSA include atherosclerotic changes with occlusion and thrombosis, occurring in about 7%-9% of cases [2,4]. Our patient's presentation with an exceptionally long-segment thrombosis measuring approximately 33 cm in the right PSA not only underscores the severity of the disease but also introduces significant technical challenges for surgical management.

### Management strategies

The management of PSA is highly individualized dictated by the anatomical subtype and the severity of occlusive disease [14].

It has been recommended that asymptomatic PSA without an aneurysm does not require intervention, but regular follow-up with duplex ultrasound is necessary due to the high incidence of complications [5,14].

Intervention treatment is often necessary for symptomatic PSAs, particularly those causing ischemia or associated with aneurysms [5,15]. Treatment involves either surgical procedures or endovascular interventions. Surgical options include interposition grafting and femoropopliteal or ilio-popliteal bypass. Endovascular methods, such as stent placement and coil or plug embolization, are becoming minimally invasive alternatives. The decision between open and endovascular repair is influenced by local expertise, anatomical suitability, and patient characteristics [1,15].

For atherosclerotic disease, percutaneous transluminal angioplasty (PTA) and stenting has been shown to be a successful alternative to surgery for focal stenosis or short occlusions [16]. However, for an extensive thrombus in an aberrant vessel, such as the 33 cm occlusion seen in our patient, open repair via femoropopliteal or ilio-popliteal bypass is often considered the most durable option [6,15]. Due to the extensive thrombosis in our patient, he was referred to a specialized vascular center for bypass surgery consideration. The treatment approach involved a right-sided ilio-popliteal bypass using a synthetic PTFE graft, as the native SFA was hypoplastic and unsuitable for revascularization. Following the procedure, the patient showed significant clinical improvement with the restoration of palpable distal pulses and a warm right extremity. The capillary refill time normalized to <2 seconds. At the 2-week follow-up, the patient reported complete resolution of rest pain and was able to ambulate without claudication. This short-term outcome underscores the effectiveness of surgical bypass in managing long-segment PSA thromboses where endovascular intervention is contraindicated [6,15]. The patient remains under regular duplex ultrasound surveillance to monitor the patency of the graft and the asymptomatic contralateral PSA [6,15].

## Conclusion

Asymmetrical bilateral PSA is a rare but critical differential diagnosis for lower limb ischemia. CTA remains the gold standard for preoperative mapping to identify the degree of SFA hypoplasia and PSA dominance. Failure to recognize this anatomy can lead to iatrogenic injury or inappropriate surgical planning.

## Ethical statement

This case report has been prepared in accordance with ethical principles related to human subject research. Written informed consent was obtained from the patient for the publication of their medical data and clinical images. The patient’s identity remains completely confidential.

The authors sincerely thank the patient and their family for their kind cooperation and for providing consent to publish this rare case.

This study was conducted in accordance with the ethical standards of the institutional research committee and with the 1975 Helsinki Declaration and its later amendments.

## Declaration of generative AI and AI-assisted technologies in the writing process

During the preparation of this work, the author(s) used a generative AI service in order to improve grammar, fluency, and academic writing style. After using this tool/service, the author(s) reviewed and edited the content as needed and take(s) full responsibility for the content of the publication.

## Authorship contributions

The authorship for the manuscript, ``*Asymmetrical Bilateral Persistent Sciatic Artery complicated with Right Limb Thrombosis: A Rare CTA Finding*'' involves 2 contributors: Amir Hossein Mahdian Dehkordi and Mohsen. Ghazi Soltani. *Amir Hossein Mahdian Dehkordi (Corresponding Author)* was responsible for the key intellectual and drafting components of the work. His primary contributions include, Conception and Design of the study, Analysis and Interpretation of the data, Writing the Manuscript, Critical Revision of the manuscript, Agreement to be Accountable and Approval of the Manuscript. Overall Responsibility: He assumes overall responsibility and guarantees the scientific integrity of the work as a whole. Beyond the works explicitly listed, the Corresponding Author is the designated authority to submit the form, manuscript, figures, tables, and all other accompanying files. He is also authorized to communicate with the Editorial Office and the Publisher regarding the submission, peer-review, revision, and publication of the manuscript on behalf of the authors., *Mohsen. Ghazi soltani (Co-author)* provided significant support in diagnosing and assessments of the patient, providing CTA images and analytical stages of the research. The co-author's key contributions include: Data Collection, Conception and Design of the study, Analysis and Interpretation of the data, Agreement to be Accountable and Approval of the Manuscript. Both authors have confirmed that the manuscript is completely original and have certified that they have no competing interests. The work did not receive any specific grant from funding agencies, and the submission is not a clinical trial.

## Patient consent

Written informed consent was obtained from the patient for the publication of this case report and any accompanying images. The authors confirm that the physical, signed consent form is retained by them and is available for review upon request, in compliance with the journal’s policy and ethical guidelines.
